# A novel way to synthesize pantothenate in bacteria involves β‐alanine synthase present in uracil degradation pathway

**DOI:** 10.1002/mbo3.1006

**Published:** 2020-02-29

**Authors:** Mariana López‐Sámano, Luis Fernando Lozano‐Aguirre Beltrán, Rosina Sánchez‐Thomas, Araceli Dávalos, Tomás Villaseñor, Jorge Donato García‐García, Alejandro García‐de los Santos

**Affiliations:** ^1^ Programa de Ingeniería Genómica, Centro de Ciencias Genómicas Universidad Nacional Autonoma de México Cuernavaca Morelos México; ^2^ Departamento de Bioquímica Instituto Nacional de Cardiología “Ignacio Chávez” Tlalpan México; ^3^ Departamento de Medicina Molecular y Bioprocesos Instituto de Biotecnología UNAM Cuernavaca México

**Keywords:** β‐alanine, pantothenate, CoA, comparative genomics, uracil degradation, pantothenate, vitamin

## Abstract

Pantothenate is an indispensable vitamin precursor of the synthesis of coenzyme A (CoA), a key metabolite required in over 100 metabolic reactions. β‐Alanine (β‐ala) is an indispensable component of pantothenate. Due to the metabolic relevance of this pathway, we assumed that orthologous genes for ß‐alanine synthesis would be present in the genomes of bacteria, archaea, and eukaryotes. However, comparative genomic studies revealed that orthologous gene replacement and loss of synteny occur at high frequency in *panD* genes. We have previously reported the atypical plasmid‐encoded location of the pantothenate pathway genes *panC* and *panB* (two copies) in *R. etli* CFN42. This study also revealed the unexpected absence of a *panD* gene encoding the aspartate decarboxylase enzyme (ADC), required for the synthesis of β‐ala. The aim of this study was to identify the source of β‐alanine in *Rhizobium etli* CFN42. In this study, we present a bioinformatic analysis and an experimental validation demonstrating that the source of β‐ala in this *R. etli* comes from β‐alanine synthase, the last enzyme of the uracil degradation pathway.

## Graphical abstract

The aim of this study was to identify the source of β‐alanine in Rhizobium etli. We show that Rhizobium etli CFN42 synthesizes β‐alanine de novo using β‐alanine synthase instead of aspartate decarboxylase enzyme.

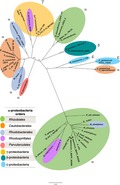

## 1 INTRODUCTION

β‐Alanine is a nonproteinogenic β‐amino acid that occurs in all living organisms. In prokaryotes, β‐ala is indispensable for the synthesis of pantothenate, the precursor of the essential cofactor coenzyme A (CoA). CoA is the source of 4'‐phosphopantetheine for fatty acid and polyketide synthesis (Leonardi & Jackowski, 2007). In eukaryotes, β‐amino acids and β‐peptides play important roles in the regulation of nutritional metabolism, immunity, and the central nervous system (Naveed Riaz, Rehman M, & Mahboob Ahmad, 2017).

The major pathway for β‐ala synthesis in *Escherichia coli* is the decarboxylation of aspartate by aspartate decarboxylase (ADC; Cronan, 1980). The ADC protein is a pyruvoyl‐dependent enzyme that is initially synthesized as a zymogen (pro‐ADC). A cleavage of pro‐ADC occurs between Gly24 and Ser25, creating the active‐site pyruvoyl moiety. Stuecker (Stuecker, Bramhacharya, Hodge‐Hanson, Suen, & Escalante‐Semerena, 2015) proposed two classes of ADC based on the type of cleavage of the zymogen (pro‐ADC). Class I of the ADC cleavage requires the MRF (Maturation Regulatory Factor) acetyl‐CoA sensor and has been found only in gammaproteobacteria. ADC Class II is an autocatalytic cleavage and is found in a wide number of bacterial phyla. Since the majority of archaea lack homologues of the *E. coli* K12 acetyl‐CoA synthesis pathway genes, the mechanism of pantothenate/CoA biosynthesis has not been completely deduced in these organisms.

The pantothenate synthesis pathway, which includes a glutamate decarboxylase (GAD) that substitutes for ADC and uses pyridoxal 5'‐phosphate (PLP) as a cofactor, was reported in archaea (Tomita, Yokooji, Ishibashi, Imanaka, & Atomia, 2014). Curiously, GAD prefers aspartate (Asp) rather than glutamate (Glu), as its substrate, although commonly GAD catalyzes the decarboxylation of Glu to γ‐aminobutyrate (GABA).

Although prokaryotes and eukaryotes have an indispensable requirement for β‐ala for the synthesis of coenzyme A (CoA), the pathways involved in its synthesis are very diverse. The uracil fermenting bacterium *Clostridium uracilicum* degrades uracil to β‐ala. Uracil or thymine is first converted to dihydrouracil. The dihydropyrimidinase enzyme catalyzes the hydration of dihydrouracil to produce *N*‐carbamoyl‐β‐ala, which is hydrolyzed to β‐ala, CO_2_, and NH_3_, by β‐ala synthase (Campbell, 1957).

The reductive degradation of pyrimidine as a source of β‐ala was supported by genetic and biochemical analyses in several bacteria, including *Clostridium uracilicum* (Campbell, 1957) and *Clostridium botulinum* (Hilton, Mead, & Elsden, 1975). Although the reductive degradation of pyrimidines has also been implicated as the de novo source of β‐ala in *E. coli* auxotrophs, lack of response to dihydrouracil indicated that in these bacteria, the major pathway for β‐ala synthesis was the decarboxylation of aspartate catalyzed by ADC.

Genschel (Genschel, 2004) performed a phyletic analysis for the occurrence of *E. coli* and human genes for in pantothenate and CoA synthesis across 47 completely sequenced genomes, 20 from the Bacteria, 16 from Archaea, and 11 from Eukarya. This study revealed a mosaic of orthologues with 20 to 70% amino acid identities. At least one protein was missing from each of the 47 analyzed genomes.

Comparative genomics using the *E. coli* pantothenate pathway genes as query against the 20 sequenced bacterial genomes revealed multiple gaps that may represent distantly related homologues due to the absence of, at least, one gene per surveyed bacterial genome (Genschel, 2004).

The *Rhizobiales* order is a heterogeneous group of Gram‐negative bacteria, taxonomically located within the alphaproteobacteria division. Some of its members are facultative diazotrophs that associate with leguminous plants to carry out symbiotic nitrogen fixation. Others are pathogens of plants or animals (Martínez‐Romero & Caballero‐Mellado, 1996). Our model is *Rhizobium etli* CFN42, which was originally isolated from bean root nodules (Martínez‐Romero, 2003). Its genome consists of a circular chromosome and six large plasmids ranging in size from 194 to 642 Kb (Gonzalez et al., 2006).

**Figure A1:**
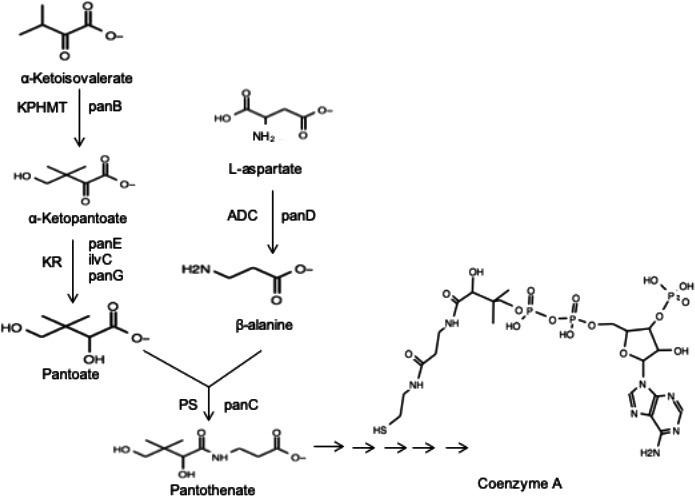
Panthotenate and CoA biosynthetic pathway

In the course of examining *Rhizobium etli* CFN42 plasmids for the presence of housekeeping genes encoding essential functions, we found that both *panC* and *panB* genes were clustered together on the 642‐kb replicon p42f. We demonstrated that both are indispensable for the synthesis of pantothenate (Villaseñor et al., 2011; Figure A1). Surprisingly, we did not find homologues of the *E. coli panD* gene in the genome of *R. etli* CFN42. Since strain CFN42 grows in minimal medium without exogenous pantothenate or β‐ala, it was assumed that it is a pantothenate prototroph.

*Agrobacterium fabrum* C58 (formerly *A. tumefaciens* C58)*,* a plant pathogen that induces tumors in numerous plants, was the only member of the *Rhizobiales* order included in Genschel's study. According to this analysis, *A. fabrum C58* lacks ketopantoate reductase (KPR, EC 1.1.1.1.169) but has a putative ADC detected by a BlastP search. We performed BlastP searches in order to gain insight on the presence or absence of ADC in the genomes of rhizobial reference strains.

Several questions arise from the presence or absence of ADC in *R. etli* CFN42. Is the absence of ADC an exclusive characteristic of strain CFN42 or is it a widespread characteristic of the *Rhizobiales* order or perhaps the alphaproteobacteria?

The aim of this work was to identify the enzyme that synthesizes β‐ala and replaces the function of ADC, allowing *R. etli* CFN42 to be a β‐ala prototroph. We also performed an in silico analysis of the alphaproteobacteria group to understand the occurrence, diversity, and evolution of the enzymes involved in β‐ala synthesis.

## 2 MATERIAL AND METHODS

### 2.1 Bacterial strains, media, and growth conditions

The characteristics of the bacterial strains and plasmids used in this study are listed in Table 1. Bacterial growth was started from glycerol stocks (20%, stored at −70°C) propagated in plates of PY‐rich medium (per L, 5 g peptone, 3 g yeast extract, 1 ml of CaCl_2_ and 15 g agar). *Rhizobium* strains were grown at 30°C in three different media: (a) PY‐rich medium, (b) chemically defined mineral medium (MM), and (c) chemically defined mineral medium plus 1 μM calcium pantothenate (MMP) or 1 μM β‐ala, added from filter sterilized stocks. Base MM containing 10 mM succinate as carbon source, 10 mM NH_4_Cl as nitrogen source, 1.26 mM K_2_HPO_4_, and 0.83 mM MgSO_4_ was adjusted to pH 6.8 and sterilized. After sterilization, the following components were added to the final concentration indicated: 1.49 mM CaCl_2_2H_2_O (autoclaved separately), 0.0184 mM FeCl_3_6H_2_O, 10 μg/ml biotin, and 10 μg/ml thiamine (all filter sterilized). MMP contains the same components plus 1 μM calcium pantothenate. Rhizobia strains were grown at 30°C for 20 hr in PY medium. *Escherichia coli* K12 MG1655 and *E. coli* BL21 (DE3) were used for cloning and to express the *R. etli* β‐alanine synthase, respectively. *E. coli* strains were grown at 37°C for 20 hr in Luria–Bertani (LB) medium (1% tryptone, 0.5% yeast extract, 0.5% NaCl, pH 7.2).

**Table 1:** Bacterial strains and plasmids used in this study

	Relevant Genotype	References
*Rhizobium etli* strains		
CFN42	Wild type, Nalr	Segovia, Young and Martinez Romero 1993
CFNX186	CFN42 cured of plasmid p42f; Nalr	Brom et al., 1992
CFN42	CFN42 pfΔ308−637	Brom et al., 1992
CFN42 RHE_CH02599‐	CFN42 RHE_CH02599::pK18mob Kmr	This study
CFN42 amaB‐	CFN42 amaB::pK18mob Kmr	This study
CFN42 amaB‐/amaB *R. etli*	CFN42 amaB::pK18mob/complemented with amaB into pFAJ1708 Tc	This study
CFN42 amaB‐/aam *R. etli*	CFN42 amaB::pK18mob/complemented with aam into pSRK Gm	This study
CFN42 amaB‐/amaB *A. fab*	CFN42 amaB::pK18mob/complemented with amaB/* A. fab* into pFAJ1708 Tcr	This study
CFN42 amaB‐/panD *A. fab*	CFN42 amaB::pK18mob/complemented with panD/* A. fab* into pFAJ1708 Tcr	This study
CFN42 amaB‐/bioa *A. fab*	CFN42 amaB::pK18mob/complemented with bioA/*A. fab* into pFAJ1708 Tcr	This study
CFN42 amaB‐/panD *E. coli*	CFN42 amaB::pK18mob/complemented with panD/*E. coli* into pFAJ1708 Tcr	This study
CFN42 amaB‐	CFN42 amaB::pK18mob/complemented with pFAJ1708 Tc	This study
*Rhizobium tropici*		
CIAT 899	CIAT 899 aam::pK18mob Kmr	This study
CIAT 899	CIAT 899 gabt::pK18mob Kmr	This study
*Agrobacterium fabrum C58*		
fabrum C58	panD::pK18mob Kmr	This study
fabrum C58	bioA::pK18mob Kmr	This study
fabrum C58	amaB::pK18mob Kmr	This study
*Escherichia coli and plasmid*		
K−12 substr. MG1655 ΔpanD	MG1655 ΔpanD::Kan	This study
K−12 substr. MG1655 ΔpanD/panD *A. fab*	MG1655 ΔpanD::Kan/panD *A. fab* into pUC19 Cbr	This study
K−12 substr. MG1655 ΔpanD/amaB *A. fab*	MG1655 ΔpanD::Kan/amaB *A. fab* into pUC19 Cbr	This study
K−12 substr. MG1655 ΔpanD/bioA *A. fab*	MG1655 ΔpanD::Kan/bioA *A. fab* into pUC19 Cbr	This study
K−12 substr. MG1655 ΔpanD/amaB *R. etli*	MG1655 ΔpanD::Kan/amaB *R. etli* into pUC19 Cbr	This study
K−12 substr. MG1655 ΔpanD/aam *R. etli*	MG1655 ΔpanD::Kan/aam *R. etli* into pUC19 Cbr	This study
K−12 substr. MG1655 ΔpanD/RHE_CH02599	MG1655 ΔpanD::Kan/RHE_CH02599 *R. etli* into pUC19 Cbr	This study
K−12 substr. MG1655 ΔpanD	MG1655 ΔpanD::Kan/complemented with pUC19 Cbr	This study
DH5α	Host for recombinant plasmids; Nalr	
pK18mob	pK18, derivative mob; Kmr	Schäfer et al. 1994
pUC19	Cloning vector Cbr	
pSRK	pBBRMC−5‐derived expression vector lac promoter, lacIq, lacZ α+, Gmr	Khan, Gaines, Roop and Farrand, 2008
pFAJ1708	Broad Host range cloning vector, Tcr	
pETSUMO	Protein and Peptide Expression System; His Tag (6x), SUMO Tag; Kmr	Hanington, Barreda and Belosevic, 2006
*Escherichia coli* BL21(DE3)	Host for recombinant plasmids;	Thermo fisher Scientific
BL21(DE3) AmaB	pETSUMO with AmaB recombinant protein	
BL21(DE3) pETSUMO	pETSUMO empty vector	

### 2.2 DNA manipulations

Standard techniques were used for plasmid and total DNA isolation, restriction digests, ligations, transformations, and agarose gel electrophoresis (Sambrook, Fritsch, & Maniatis, 1989). Plasmid mobilization from *E. coli* to *Rhizobium* was done by conjugation on PY plates at 30°C by using overnight stationary phase cultures. Donors (*E. coli* DH5) and recipients (*R. etli* CFN42 wild‐type and mutant strains) were mixed at a 1:2 ratio, and suitable markers were used for transconjugant selection.

### 2.3 Analysis for the occurrence of 12 proteins involved in pantothenate synthesis and phylogenetic analysis of putative ADC enzymes found in alphaproteobacteria

We selected 204 alphaproteobacteria to analyze for the presence and absence of 12 proteins related to the pantothenate synthesis and transport. The protein FASTA files (faa) for each of the genomes were downloaded from the RefSeq NCBI database. Protein sequences with an expectation value (E) of 10^−3^ or less were considered as putative homologues. We used Proteinortho v5.15 to obtain the clusters of orthologous proteins from the 204 protein FASTA files. Next, we used the Pfam v31.0 database to determine which protein ortho clusters represent the 12 proteins of interest analyzed in this work. The proteins we searched for were PYD1, PYD2, PYD3, GAD, KPHMT, PS, ADC, KPR, MRF, KAR, Aam, and GabT. Finally, we determined which alphaproteobacteria were represented in each protein cluster (Table A2).

For phylogenetic analysis, we used the Pfam v31.0 database to determine which proteinortho clusters represent the ADC proteins. A total of 37 homologues belonging to alphaproteobacteria sequences were tested with a group of nine external sequences listed in Table A3 and were aligned against Muscle v3.8.31.

**Figure A2:**
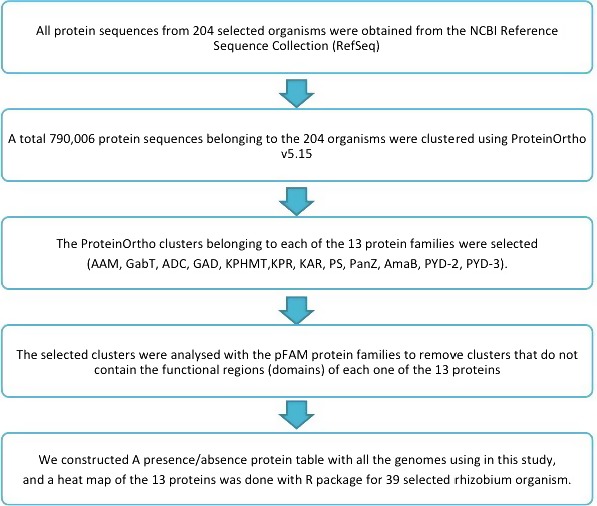
Flow diagram of the bioinformatics search methodology

The resulting data set containing 46 putative ADC homologues was used to infer the evolutionary relationships. We used ProtTest3 v3.4.2 for the evolutionary model, and the best result was LG + G model, using amino acid alignment. The phylogenetic analysis was performed with PhyML v3.3.20170530 under (‐d aa ‐m LG ‐a e ‐o ltr) parameters **(**Figure A2).

### 2.4 Cloning and sequence analysis of *amaB* gene, mutants, and complemented mutant

The *ama*B (RHE_CH03290) gene was overexpressed in *E. coli* DH5‐alpha. The coding region of the *amaB* gene was amplified from the genomic DNA of *R. etli* CFN42 by PCR. The amplified fragment was inserted into the pET‐SUMO expression vector (Ni‐NTA Purification System; Sigma‐Aldrich). After confirming the absence of mutations, the plasmid was introduced into *E. coli* strain BL21 (DE3). Primer set list is in Table A1 **.**

### 2.5 Overexpression and purification of wild‐type β‐ureidopropionase AmaB

The transformant BL21(DE3) strain was grown in LB medium supplemented with 100 mg/ml of carbenicillin. A single colony was transferred into 10 ml of LB medium with carbenicillin at the above‐mentioned concentration in a 100‐ml flask. This culture was incubated overnight at 37°C with shaking. Five hundred milliliters of LB medium with 100 mg/ml of carbenicillin was inoculated with 5 ml of the overnight culture in a 1‐liter flask. After 3 hr of incubation at 37°C with vigorous shaking, the optical density at 600 nm (OD600) of the culture was 0.3–0.5. For induction of β‐alanine synthase gene expression, isopropyl‐β‐D‐thiogalactopyranoside (IPTG) was added to a final concentration of 0.1 mM and incubation was continued at 30°C for an additional 6 hr.

The cells were collected by centrifugation (8,000 × *g*, 4°C, 10 min), washed twice in wash buffer (2.5 M NaCl, 250 mM NaH_2_PO_4_, 20 mM imidazole pH 8.0), and resuspended in 50 ml of the same buffer. The cells were disrupted by sonication using a UP200S ultrasonic processor, in ice for four periods of 15 s at pulse mode 0.5 and 40% sonic power. The cell debris was pelleted by centrifugation (8,000 × *g*, 4°C, 10 min), and the supernatant was applied to a 2‐ml column of nickel metal‐affinity resin (Ni‐NTA Purification System; Sigma‐Aldrich) and β‐ureidopropionase purified as recommended by the manufacturer. The purified enzyme was dialyzed against 20 mM sodium phosphate buffer, pH 8.0, and stored at 4°C.

### 2.6 Enzyme assays

The standard enzymatic reaction was carried out with purified AmaB at a final concentration of 1 mg/ml along with 125 mM 3‐ureidopropionic acid and 10 mM MgCl_2_ dissolved in 100 mM sodium phosphate buffer, pH 8.0, in a 3 ml reaction volume (Martinez‐Gomez et al., 2008). The reaction mixture was incubated at 30°C for 60 min, with the apoenzyme preincubated (1 hr) at 4°C with 2 mM of NiCl_2_, and 500 µl samples were stopped for every 15 min, by the addition of 50 µl of 3% TCA. After centrifugation, the presence of β‐ala in the resulting supernatants was estimated by high‐performance liquid chromatography (HPLC).

### 2.7 Determination of β‐ala by HPLC/fluorescence

Determination of β‐ala was carried out by HPLC coupled to a Multi γ–fluorescence detector (Waters 1525/2475) using a reverse‐phase C‐18 Spherisorb ODS2 column of 5 μm particle size and 150 × 4.6 mm (Waters; García‐García, Peña‐Sanabria, Sánchez‐Thomas, & Moreno‐Sánchez, 2018). Enzymatic reactions were stopped with perchloric acid (3% v/v) at the indicated times and immediately frozen in liquid nitrogen and kept at −70°C. The acidic supernatants were neutralized with 3 M KOH/0.1 M Tris and centrifuged to remove KClO_4_. Supernatant was recovered and used for β‐ala determination by derivatization with 37 mM ortho‐phthalaldehyde (OPA). β‐ala and α‐alanine standards (Sigma‐Aldrich, Saint Louis, MO, USA) were used for identifying of chromatographic peaks.

## 3 RESULTS

### 3.1 Orthologues of the canonical L‐aspartate‐α‐decarboxylase enzyme are predominantly absent in α‐proteobacteria

A previous study on ADC phylogeny and amino acid conservation analyses revealed that ADCs are present in γ‐proteobacterial genomes and most maintain the *panCBD* synteny (Stuecker et al., 2015). We noticed the absence of the *panCBD* gene cluster while functionally characterizing *panC* and *panB* in rhizobia (Villaseñor et al., 2011). In the present study, BlastP and Psi Blast searches using ADC from *E. coli* and *A. fabrum* C58 as query revealed the absence of ADC homologues in *R. etli* CFN42 and other reference strains (Table A2).

To generalize the absence of ADC homologues in α‐proteobacteria, we assessed the occurrence of putative ADCs in the proteome of 204 alphaproteobacteria, 84 rhizobia and 120 members of seven families of alphaproteobacteria (Table A2). The complete proteome of each bacterium was obtained from the NCBI reference sequence collection (RefSeq) and clustered with Proteinortho v5.15, a large‐scale Blast‐based orthology detection tool (Lechner et al., 2011; Figure A2). This analysis only showed 37 putative ADCs from 204 α‐proteobacteria genomes.

### 3.2 Unrooted maximum‐likelihood‐based tree inferred from the alpha‐ and gammaproteobacteria ADCs revealed high divergence among them

An important characteristic of the alphaproteobacteria is its genome plasticity, which allows different genome rearrangements, including deletions or duplications (Prell & Poole, 2006; Tiwari & Lata, 2018). We made a phylogenetic analysis to get a wider view of the evolutionary relationship among the ADCs from the α‐, β‐, γ‐, and ε‐proteobacteria (Table A3).

**Figure 1:**
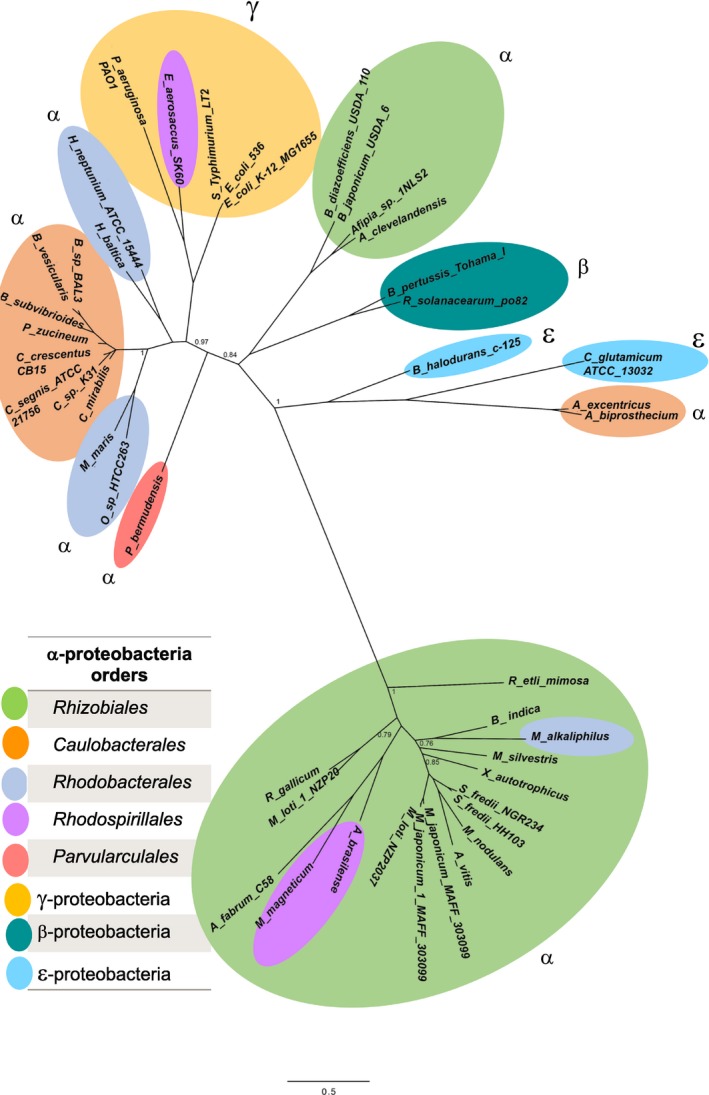
The putative ADCs of alphaproteobacteria found in our occurrence analysis. A maximum‐likelihood phylogenetic tree inferred from a subset of 204 genomes, where we extracted only 37 ADCs. The tree shows a monophyletic clade of proteins distantly related to those from γ‐proteobacteria and other α‐proteobacteria

The resulting maximum‐likelihood‐based tree is shown in Figure 1, and the data set is presented in Table A3. To determine if this ADC phylogeny maintains the coherence of species phylogeny, it was compared to the previously reported species trees performed by the Bayesian analysis of 104 concatenated alignments (Williams, Sobral, & Dickerman, 2007) and with the most recent robust species tree; this was done under the maximum‐likelihood framework with a data set of 200 single‐copy and conserved genes for the alphaproteobacteria (Muñoz‐Gómez et al., 2019).

The majority of ADCs belonging to the *Rhizobiales* order were grouped in a single cluster (Figure 1, green oval bottom). Unexpectedly, we found two ADCs from *Bradyrhizobium japonicum* and *Afipia* sp close to γ‐ and β‐proteobacteria (Figure 1, upper green oval). Two ADCs of the *Rhodospirillales* order *(Azospirillum brasilense and Magnetospirillum magneticum)* were located as orthologues of *Rhizobiales* (Figure 1, purple oval). The ADC from *Maritimibacter alkaliphilus* was located within the *Rhizobiales* order, whereas in the species tree, *M. alkaliphilus* belongs to the *Rhodobacterales* order (Muñoz‐Gómez et al., 2019).

This heterogeneous cluster of *Rhizobiales* ADCs links through a long branch with remote orthologues from class alphaproteobacteria belonging to the following orders: *Rhizobiales* (*B. japonicum* and *Afipia)*, *Caulobacterales* (*C. crescentus*), *Rhodobacterales* (*Hyphomonas neptuniou* and *Hirschia baltica*), and *Parvularculales* (Parvularcula bermudensis γ‐proteobacteria (outgroup, *E. coli*, *Salmonella, Pseudomonas aeruginosa*), β‐proteobacteria (*B. pertussis* and *R. solanacearum*), and ε‐proteobacteria (*Corynebacterium glutamicum*)).

### 3.3 Presence, absence, duplications, and functional redundancy of the six *pan* genes involved in pantothenate synthesis

In addition to ADC, the 84 rhizobial genomes were surveyed for the presence of the enzymes that catalyze pantothenate synthesis. This revealed that the KPHMT (ketopantoate hydroxymethyl transferase, PanB) is highly conserved in the *Rhizobiales* order and was absent in only 8.4% of the analyzed genomes. The genera lacking KPHMT were *Bradyrhizobium* sp ORS 278, *Candidatus liberobacter* (4 strains), and *Hoeflea* (2 species). KPHMT was predicted to be present in the other members of *Rhizobiales,* which have a diversity of habitats. Two copies of this enzyme were present in 17.8% of rhizobia, mostly in the *Rhizobium* and *Sinorhizobium* species. Three copies of KPHMT were found in 4.8% of the *Rhizobiales* order: three *Mesorhizobium* species and one in *Rhizobium leucaenae* (Table A2).

The next step in the pathway is the reduction of α‐ketopantoate to produce pantoate. Two enzymes can perform this reduction: KPR (α‐ketopantoate reductase, PanE) was found in only 57% of the rhizobial genomes, while KAR (acetohydroxy acid reductoisomerase, ilvC) was present in 95.2% of the genomes (Table A2). Most human, plant, and mammalian pathogens have lost the KPR enzyme. Interestingly, *Candidatus* genera lacked both KAR and KPR enzymes in their genomes.

In the last step of the pathway, pantothenate synthetase (PS, PanC) catalyzes the ATP‐dependent condensation of D‐pantoate with β‐ala to form pantothenate. This enzyme was absent in 7.1% of the surveyed genomes, some of which belong to parasites such as *Hoeflea* and *Candidatus* (Table A2). Strains with a single copy were found in 88% of the analyzed rhizobia genomes. Two genes were found in 4.7% of *Mesorhizobium* and *Bradyrhizobium* species.

The occurrence of putative ADC enzymes is shown in Table 2. An ADC encoding gene was present in 19% of the genomes, and two strains had a second copy of ADC in their genomes. *Mesorhizobium japonicum* MAFF303099 had one in a chromosome and the other one in a plasmid, and *M. loti* NZP 2037 had both in a chromosome.

**Table 2:** Occurrence* of pantothenate synthesis genes on Rhizobiales order

Gene	Enzyme	Occurrence (%)
*panD*	ADC	19.04
*panM*	MRF	21.42
*panB*	KPHMT	91.66
*panE*	KPR	57.14
*ilvC*	KAR	95.23
*panC*	PS	92.85

Note

* The percentage was calculated based on the number of rhizobia bacteria that covered the sample (*n* = 84).

The search for MRF (Maturation Regulatory Factor, *panM*) homologues revealed that 78.5% of the genomes lacked an MRF homologue; 15.4% had one copy and 6% encoded two copies. However, only five genomes coded for both ADC and MRF *(A. fabrum* C58, *Bradyrhizobium japonicum* USDA11, *R. etli bv mimosa* str. Mim1, *Rhizobium gallicum,* and *Sinorhizobium fredii* HH130; Table A2).

Our results showed that only 16 of the 84 analyzed genomes (19.04%) encoded the complete pantothenate pathway. Of the 68 genomes with gaps in the pathway, the predominant deficiencies were a lack of ADC in 80.95% of the genomes and the absence of both ADC and KPR in 38%.

### 3.4 *Rhizobium etli* CFN42 is a pantothenate prototroph

**Figure 2:**
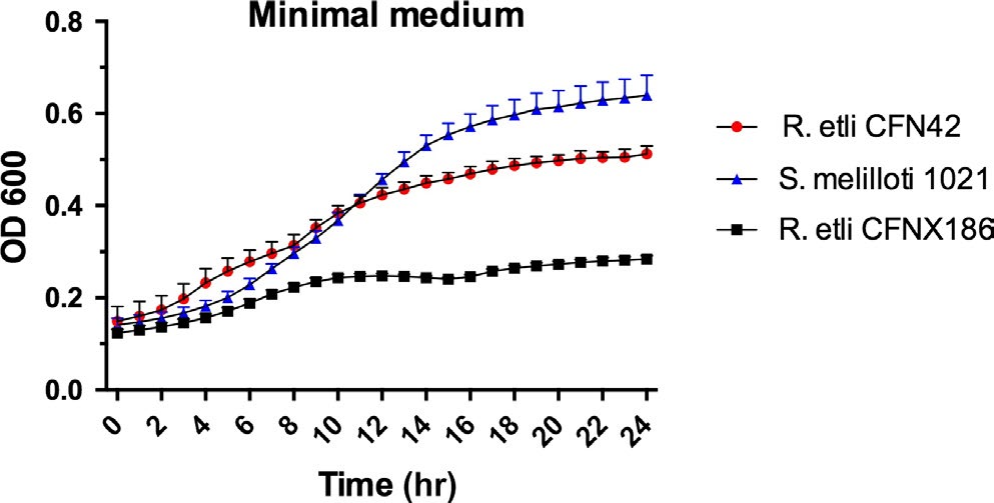
Growth test for prototrophy of wild‐type *Rhizobium etli* CFN42, its p42f‐cured derivative CFNX186, and in wild‐type *Sinorhizobium meliloti* 1,021. Tested in minimal medium without β‐alanine or pantothenate

The model of pantothenate synthesis established in *E. coli* (Cronan, 1980; Leonardi & Jackowski, 2007) indicates that the enzymes missing in rhizobia should cause auxotrophy. Growth assays were done in liquid chemically defined medium with *R. etli* CFN42 (lacks *panD*) and *Sinorhizobium meliloti* 1,021 (lacks *panD* and *panE*) wild‐type strains, and an *R. etli* CFN42 plasmid p42f‐cured strain (CFNX186) that is defective for growth in chemically defined medium without pantothenate. We found that the wild‐type strains were able to grow through three subcultures in minimum medium without β‐ala or pantothenate. This shows that even with the absence of *panE* and/or *panD,* rhizobia are still able to synthesize β‐ala and pantothenate (Figure 2). This prototrophy contrasts with the auxotrophy exhibited by *R. etli* CFNX186, which lacks *panC* and *panB,* as well as plasmid p42f (Brom et al., 1992).

### 3.5 Occurrence analysis revealed different pathways that would replace ADC in rhizobia

**Figure 3:**
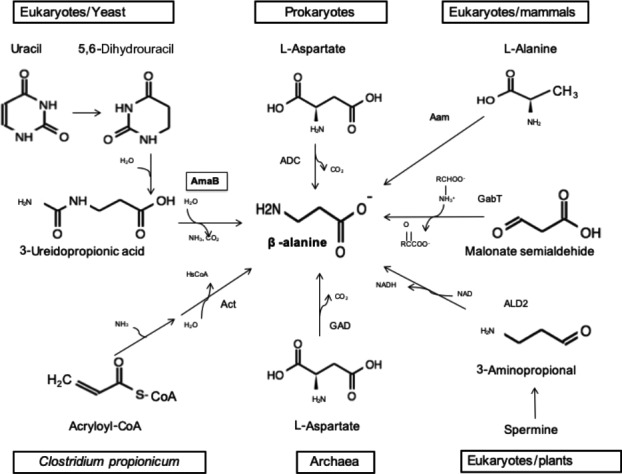
β‐Alanine biosynthesis in different domains of life. (AmaB) β‐alanine synthase; (ADC) 1‐aspartate decarboxylase; (Aam) 2,3‐aminomutase; (gabT) 4‐aminobutyrate transferase; (ALD2) amine oxidase; (GAD) glutamate decarboxylase; (Act) β‐alanyl‐CoA:ammonia lyase

To identify which enzyme(s) might be responsible for the synthesis of β‐ala, we performed bioinformatic analyses of 204 alphaproteobacterial genomes to find possible pathways or genes that could potentially produce this metabolite. Based on a literature search, we selected six genes of interest that encode enzymes of the pyrimidine degradation pathway (AmaB, Dht, PyrD), glutamate decarboxylase (GAD), and the Aam and GabT transaminases (Figure 3).

It is believed that β‐ala synthesis in bacteria results only through decarboxylation of aspartate by ADC (Cronan, Littel, & Jackowski, 1982; David & Lichstein, 1950). Other ways of producing β‐ala exist in eukaryotes. Two routes occur in fungi: *Saccharomyces cerevisiae* produces β‐ala by the degradation of spermine (White, Gunyuzlu, & Toyn, 2001), and *Schizosaccharomyces pombe* and *Saccharomyces kluyveri* obtain it from uracil degradation (Lundgren, Gojković, Piškur, & Dobritzsch, 2003; Table A2, Figure 3).

The pyrimidine degradation pathway involves three enzymatic steps from uracil to produce β‐ala, CO_2_, and NH_3_ (Campbell, 1957). In the final step of the pathway, β‐ala synthase (AmaB) uses *N*‐carbamoyl‐β‐alanine as substrate. In rhizobia, the in vitro activity of AmaB has been detected in *A. fabrum* C58 and *S. meliloti* 1021. The authors showed the production of β‐ala from 3‐ureidopropionic acid in vitro, in the last step of the pathway (Martínez‐Rodríguez, Martínez‐Gómez, Rodríguez‐Vico, Clemente‐Jiménez, & Las Heras‐Vázquez, 2010). In archaea, β‐ala can be synthesized by a GAD that uses Asp as a substrate. In these studies, it was shown that two enzymes annotated as GADs had higher affinity for Asp than for Glu and they demonstrated the in vitro activity of the enzymes in *Methanocaldococcus jannaschii* and *Thermococcus kodakarensis* (Tomita et al., 2014; Wang, Xu, & White, 2014).

We also included in the study two transaminases that in bacteria, insects, and mammals produce β‐ala in a single‐step reaction. The first one, Aam, acts on L‐alanine and 3‐oxopropanoate to produce pyruvate and β‐ala (Dalluge, Liao, Gokarn, & Jessen, 2005; Yun, Lim, Cho, & Kim, 2004). The second, GabT, performs a transamination of malonate semialdehyde and L‐glutamate (Nanaya, Hidenori, Keiko, tatsuhiko, Ikeda, & Takao, 1982; Wilding, Peat, Newman, & Scott, 2016).

In summary, our bioinformatic analysis showed that two transaminases and the pyrimidine degradation pathway were encoded in the *R. etli* CFN42 genome. We did not find any candidate genes for ADC or GAD, nor a complete polyamine degradation pathway.

### 3.6 AmaB functionally complements strains lacking ADC

In our study, we tested the function of different genes in *R. etli*, by inactivating those that encode two transaminases (Aam and GabT) and the *amaB* gene for pyrimidine degradation (Table A1). Following with the canonical decarboxylation pathway, we found a putative ω‐amino acid decarboxylase that was different from the ADC and GAD enzymes. The genes were interrupted using a suicide plasmid, and the resulting mutants were tested for growth in defined medium without β‐ala or pantothenate. From this screening, we found that the *amaB* mutant was auxotrophic for β‐ala, while inactivation of the other genes caused no growth deficiency (data not shown).

**Figure 4:**
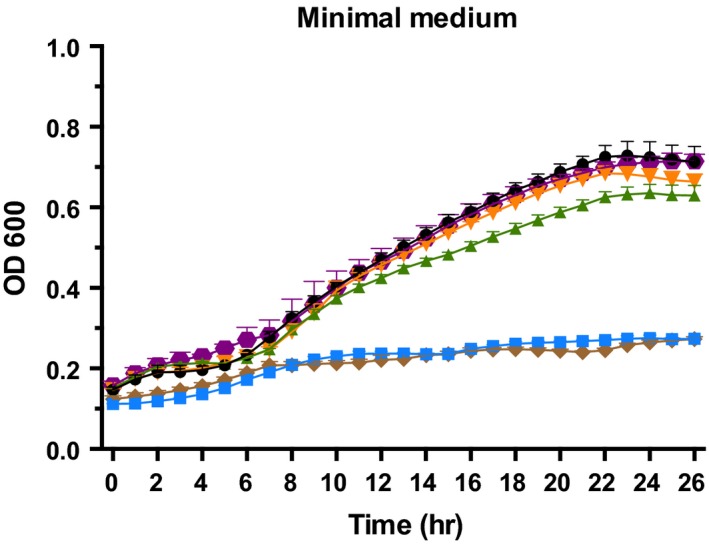
Growth in minimal medium of the *Rhizobium etli* CFN42 wild‐type (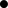) strain and its derivative complements. *R. etli* CFN42 *amaB* mutant (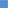); CFN42 *amaB/amaB^+^* of *R. etli* CFN42 (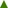); CFN42 *amaB/amaB^+^* of *A. fabrum* C58 (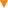); CFN42 *amaB* complemented with 1 µM of β‐alanine (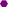); CFN42 *amaB*/pFAJ1708 empty vector (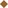)

*amaB* (RHE_CH03290) is a chromosomal gene annotated as β‐alanine synthase. It belongs to the pyrimidine degradation pathway and transforms 3‐ureidopropionic acid to β‐ala, CO_2_, and ammonia. We disrupted this gene in *R. etli* CFN42 and grew the ReAM‐1 (*amaB^‐^*) mutant in mineral medium (MM) without β‐ala or pantothenic acid. The mutant was deficient in growth, indicating a β‐ala auxotrophy, and its growth was restored by exogenous β‐ala or by introducing the *amaB* gene in a plasmid (Figure 4).

Similarly, the mutant was complemented with a plasmid‐borne copy of the *amaB* gene from *A. fabrum* C58. The product of this gene has been shown to have β‐alanine synthase activity in vitro (Martinez‐Gomez et al., 2008).

### 3.7 Purified AmaB produces β‐ala from 3‐ureidopropionic acid in vitro

**Figure A3:**
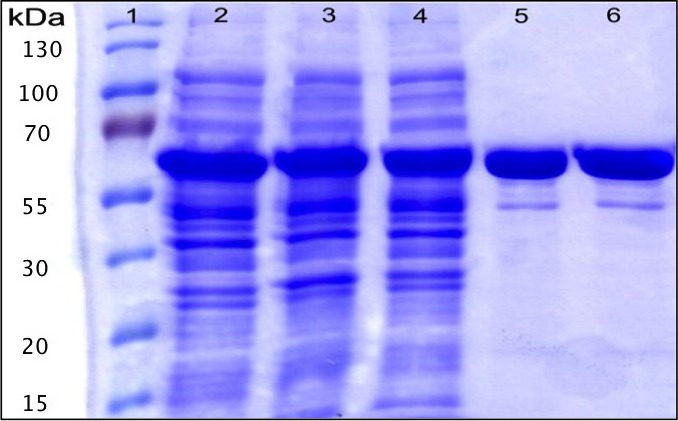
SDS‐PAGE analysis of the His6‐3‐Ureidopropionase produced by *Escherichia coli *harboring pETSUMO. Lane 1: Protein size marker, lane 2: cell extract after 6h of induction at 30.C, lane: 3 insoluble fraction, lane 4: soluble fraction after induction, lanes 5‐6: elution fraction, lane: 7 elution fraction after filtration by 30 kDa amikon, lane8: concentrated elution fraction with glycerol

The his_6_‐tag enzyme was purified in an immobilized nickel affinity column under native conditions and had a molecular mass of 60 kDa, consistent with β‐ala synthase (45 kDa), plus the 15‐kDa 6His‐Sumo tag (Figure A3).

**Figure A4:**
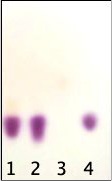
TLC enzymatic reactions plate revealed with ninhydrin. (1) Test reaction; (2) Reaction with β–ala; (3) Control reaction without enzyme; (4) β–ala standard

Enzymes of this type are characterized as metalloenzymes that use Ni^2+^ and Co^2+^ as cofactors in enzyme assays. The reaction mixture contained purified AmaB preincubated with Ni^2+^ or Co^2+^, 10 mM MgCl_2_, 100 mM sodium phosphate buffer, and 3‐ureidopropionic acid as a substrate. We initially used a TLC system with ninhydrin detection to identify the presence of β‐ala (Niederwieser et al., 1971; Figure A4). We observed enzymatic activity with both metal ions, and no product was formed in their absence.

As described below, we also performed our enzymatic assays using an HPLC system to obtain a better resolution.

### 3.8 Synthesis of β‐ala by recombinant AmaB

**Figure 5:**
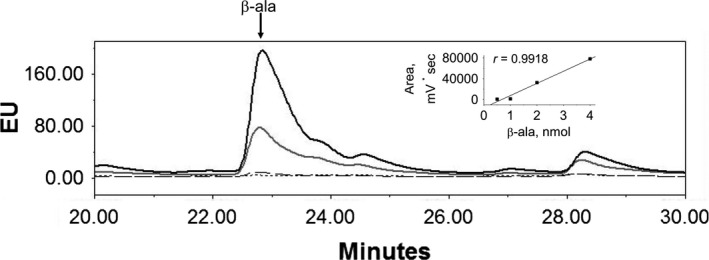
Standard of β‐ala was analyzed by HPLC/fluorescence at 0.5 (dotted line), 1 (dashed line), 2 (gray line), and 4 (black line) nmols following the protocol detailed in Material and Methods. Inset shows the linear analysis of areas from each peak

**Figure 6:**
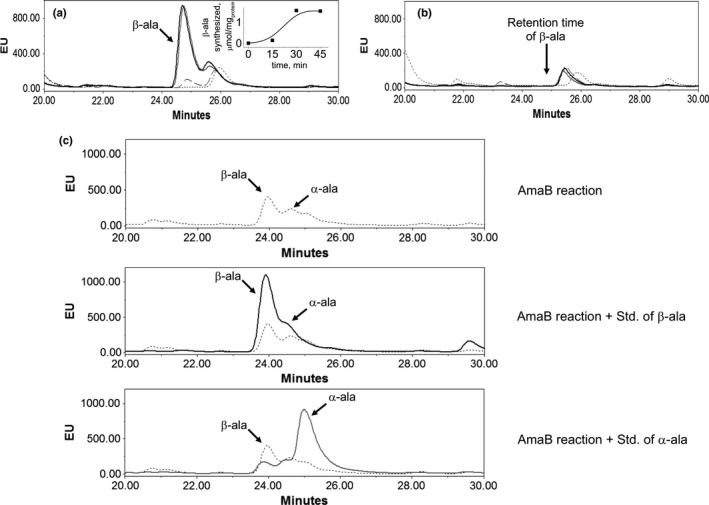
Analysis β‐alanine synthesized by AmaB. (a) Activity of recombinant AmaB, at 0 (dotted line), 15 (dashed line), 30 (gray line), and 45 min (black line). Peak of β‐ala was observed at 24.8 min. (b) Reaction without enzyme; β‐ala peak was not observed. (c) To confirm peak of β‐ala, internal standard of β‐ala (black line) and L‐ala (gray line) was added independently to neutralized AmaB reaction carried out for 15 min (dotted line)

The *R. etli* CFN42 *amaB* gene was heterologously expressed in *E. coli* strain BL21 (DE3) and recovered by Ni^2+^ affinity chromatography, as previously described (Martinez‐Gomez et al., 2008). Production of β‐ala by recombinant AmaB was analyzed by HPLC. The fluorescence response of β‐ala had a linear relation with concentration (Figure 5). The time course of recombinant AmaB activity using 3‐ureidopropionic acid as substrate and Ni^2+^ as cofactor showed that β‐ala is synthesized at a linear rate for up to 30 min (Figure 6a). β‐ala was not detected in a reaction assay without recombinant AmaB protein (Figure 6b). The standard of β‐ala overlapped with the peak of the compound synthesized by AmaB, while the α‐ala standard did not (Figure 6c). These results indicated that recombinant AmaB is able to synthesize β‐ala.

## 4 DISCUSSION

The relevance of β‐ala as a key component of pantothenate synthesis has been well established. However, the diversity of mechanisms described in bacteria and eukaryotes suggests that the synthesis of β‐ala has not been totally elucidated.

Pioneer studies performed in *E. coli* and γ‐proteobacteria defined that β‐ala was synthesized by the decarboxylation of L‐aspartate in a one‐step reaction catalyzed by ADC. The concept of a canonical one‐step decarboxylation reaction was for many years assumed to be the sole source of β‐ala in bacteria.

The genomic era facilitates the comparison of pathways among numerous species (Genschel, 2004); this bioinformatic approach helped us determine the diversity of mechanisms involved in β‐ala synthesis. In this study, we found several differences between *R. etli* (alphaproteobacteria) and *E. coli* (γ‐proteobacteria); the most intriguing was the absence of an ADC homologue in rhizobia. In previous studies, analyses of *E. coli* and other γ‐proteobacteria revealed that β‐ala was produced by the decarboxylation of aspartate by aspartate decarboxylase enzyme (ADC; Cronan et al., 1982); in several archaea, β‐ala was synthesized by a glutamate decarboxylase (GAD) able to decarboxylate both aspartate and glutamate (Tomita et al., 2014). These data confirm the relevance of one‐step decarboxylases, not only in bacteria but also in archaea.

An unusual alternative source of β‐ala synthesis is the reductive degradation of pyrimidine. This three‐step reaction was found in *Clostridium uracilicum* (Campbell, 1957) and *C. botulinum* (Hilton et al., 1975), as well as in *E. coli* strains: *E. coli* W, *E. coli* D2, *E. coli* 99–1, and *E. coli* 99–2 (Table A2); in contrast to previous studies, none of them was able to grow in the presence of dihydrouracil and β‐ureidopropionic acid (Slotnick & Weinfeld, 1956).

In bacteria belonging to the *Rhizobiales* order, little is known about the metabolism of β‐ala and pantothenate (Villaseñor et al., 2011). The occurrence analysis performed in this work indicates that our model, *R. etli* CFN42, lacks ADC and GAD, the most common one‐step reaction used in bacteria to synthesize β‐ala. We suggest that there can be functional redundancy in certain rhizobia strains. As part of our work, we constructed different single and double mutants in *A. fabrum* C58 to try to get an auxotrophic strain, but in all cases, the mutants continue to be β‐ala prototrophic (data not shown).

**Figure A5:**
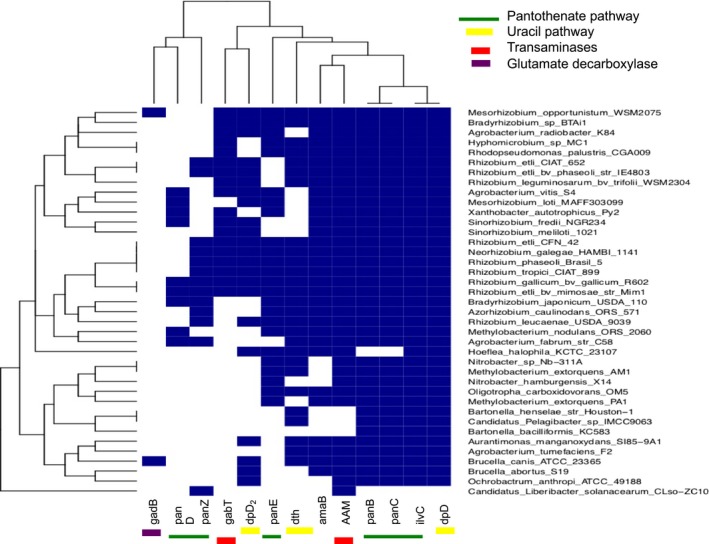
Alternatives pathways or enzymes that can act in the absent of ADC enzyme. Heat‐map constructed with 40 representative Rhizobiales genomes

Particularly for the *Rhizobiales* order, we constructed a heat map with their most representative genomes; here, we can associate the loss and prevalence of different pathways, assuming that the decarboxylation pathway is missing in most of rhizobia genomes (Figure A5).

*Sinorhizobium meliloti* and *A. fabrum C58* have been tested for production of β‐amino acids through the uracil degradation pathway because of their pharmaceutical relevance (Martínez‐Rodríguez et al., 2010). Unexpectedly, the research only showed the ability to produce β and ω amino acids in vitro; we do not know if these strains synthesize β‐ala through this pathway or if these strains have a functional redundancy with another β‐ala synthesis pathway.

As part of our occurrence analysis, we extended our work to alphaproteobacteria with 120 more genomes from seven different orders (Table A2). We found a correlation between the rhizobia order and alphaproteobacteria. In general, we observed that the pyrimidine degradation pathway (37%) and Aam transaminase (56%) are widely distributed in alphaproteobacteria, as well as in rhizobia (Table A2). We also observed that ADC and GAD enzymes are poorly represented in alphaproteobacteria, with 17% and 6.8%, respectively. This analysis suggests a strong correlation between the loss of the decarboxylation pathway and predominance of the pyrimidine degradation pathway in the *Rhizobiales* order and in alphaproteobacteria.

We also tested the activity of recombinant AmaB in vitro*,* by HPLC, to confirm the catalytic activity of this protein by showing that it produces β‐ala from 3‐ureidopropionic acid; this corroborates the presence of alternative pathway in which bacteria produce this essential metabolite.

## 5 CONCLUSIONS

Prokaryotes and eukaryotes require β‐ala to synthesize CoA; however, the source of β‐ala is quite variable even in bacteria.

For years, it has been assumed that the main source of β‐ala in prokaryotes comes from the decarboxylation of aspartate in a single enzymatic step catalyzed by ADC. This reaction was discovered in *E. coli* and has been assumed to be the main source of β‐ala in γ‐proteobacteria.

This study in *R. etli* CFN42 and other alphaproteobacteria revealed a remarkable reduction of ADC orthologs in these bacteria.

The bioinformatics and experimental analyses performed with rhizobia indicate that in these alphaproteobacteria β‐ala is synthesized through the reductive pyrimidine degradation pathway.

All these data highlight the metabolic plasticity for β‐ala and pantothenate in bacteria.

## CONFLICT OF INTEREST

None declared.

## AUTHOR CONTRIBUTIONS

Mariana Lòpez‐Sámano; Formal analysis‐Equal, investigation‐Equal, methodology‐Equal, Writing‐original draft‐Equal. Luis Fernando Lozano‐Aguirre Beltàn; Data curation‐Equal, software‐supporting, supervision supporting, validation supporting. Rosina Sánchez‐Thomas; Formal analysis ‐supporting, methodology‐supporting, validation‐supporting. Araceli Dávalos; Investigation‐supporting, methodology‐supporting, supervision‐supporting. Tomás Villaseñor; Investigation‐supporting, supervision‐supporting. Jorge Donato García‐García; Formal analysis ‐supporting, methodology‐supporting, validation‐supporting. Alejandro García‐ de los Santos: Conceptualization‐Equal, formal analysis‐Equal, funding acquisition‐supporting, investigation ‐Equal, supervision‐Equal, writing original draft‐Equal.

## ETHICS STATEMENT

None required.

## Data Availability

All 204 data used in this study were download from RefSeq public NCBI database. The ID for each of the amino acid sequences is available through tools at different websites as described in Material and Methods, or upon request from the corresponding author. The supplementary material in the text referred to as Tables A2 and A3 was uploaded in a Zenodo data repository with DOI numbers https://doi.org/10.5281/zenodo.3593441 and https://doi.org/10.5281/zenodo.3593474, respectively.

## Appendix 1

**Table A1:** Primer list

Mutant	Primer	
CFN42 RHE_CH02599‐	5′ATC CTC GAA TTC AAG GCT CTA AGC TGC C 3′	Forward
	5′ TGT GAA GGA TCC GCG CTT CAC AAA 3′	Reverse
CFN42 amaB‐	5′‐ CGT GCA GGA TCC GGA CTT CGC CTA TAA C ‐3′	Forward
	5′‐ GAG CTT AAG CTT GTC GGG TGA GCG GAT G ‐3′	Reverse
CFN42 amaB‐/amaB *R. etli*	5′‐ ATC CGC AAG CTT AAA ACC AAA GGC AAC T ‐3′	Forward
	5′‐ GAA GGT GGA TCC AAG GGT CGG ATG A ‐3′	Reverse
CFN42 amaB‐/aam *R. etli*	5′‐ CAT GAT GGA TCC GTT TGC GTT GTC CAG A ‐3′	Forward
	5′‐ CCC ATC GAA TTC GTT TTG CCG CCG AAT A ‐3′	Reverse
CFN42 amaB‐/amaB *A. fab*	5′‐ CGC CAT GGA TCC GCA ATG GCT GTT ATC T ‐3′	Forward
	5′‐ CTG CCG GAA TTC ATC CTG ATG TCT GCC T ‐3′	Reverse
CFN42 amaB‐/panD *A. fab*	5′‐ CCG ATG AAG CTT CGA CAA AGA TCG GCA A ‐3′	Forward
	5′‐ GAT GTC GAA TTC GAA CCT CTG GTC GCC T ‐3′	Reverse
CFN42 amaB‐/panD *E. coli*	5′‐ CAC CAG GAA TTC CAT CGT CTC CAG CGA A ‐3′	Forward
	5′‐ GGT GAG AAG CTT GCC GCA GGG ATA ACA A ‐3′	Reverse
BL21 (DE3) AmaB/pETSUMO	5′ATGGTGGCAGCACCAGGCGAGAACATGC‐3′	Forward
	5′‐ TCACACCACGATCTCCGCCGTCTCCACC‐3′	Reverse

Abbreviations: *R. etli, Rhizobium etli; A. fab, Agrobacterium fabrum; E. coli,* *Escherichia coli*.

**Table A2:** Occurrence analysis in alpha‐proteobacteria, represented by number of copies of each gene on strain genome.

Class	Order	Organism	Biosynthesis pathway									Gene		
			Pyrimidine degradation			Pantothenate pathway						Transaminases		Decarboxylase
			PYD3	PYD2	PYD1	KAR	KPHMT	PS	ADC	KPR	panZ	AAM	GabT	GAD
Alphaproteobacteria	*Rhizobiales*	Afipia_sp_1NLS2	1	1	1	1	1	1	1	3	0	1	0	0
		Agrobacterium_fabrum_str_C58	1	1	1	1	1	1	1	0	2	2	0	0
		Agrobacterium_radiobacter_K84	1	0	1	1	2	1	0	1	0	2	1	0
		Agrobacterium_sp_H13‐3	1	1	1	1	1	1	0	1	0	2	0	0
		Agrobacterium_tumefaciens_F2	1	1	1	1	1	1	0	0	0	2	0	0
		Agrobacterium_vitis_S4	1	0	1	1	1	1	1	1	0	1	1	0
		Aurantimonas_manganoxydans_SI85‐9A1	1	3	1	1	1	1	0	0	0	1	0	0
		Azorhizobium_caulinodans_ORS_571	1	1	1	1	1	1	0	1	1	1	0	0
		Bartonella_bacilliformis_KC583	0	0	1	1	1	1	0	0	0	0	0	0
		Bartonella_clarridgeiae_73	0	1	1	1	1	1	0	0	0	0	0	0
		Bartonella_grahamii_as4aup	0	1	1	1	1	1	0	0	0	0	0	0
		Bartonella_henselae_str_Houston‐1	0	1	1	1	1	1	0	0	0	0	0	0
		Bartonella_quintana_str_Toulouse	0	1	1	1	1	1	0	0	0	0	0	0
		Bartonella_tribocorum_CIP_105476	0	1	1	1	1	1	0	0	0	0	0	0
		Beijerinckia_indica_subsp_indica_ATCC_9039	0	1	1	1	1	1	1	0	0	1	0	0
		Bradyrhizobiaceae_bacterium_SG‐6C (A. clevelandensis ATCC49720)	1	1	1	1	1	1	1	3	0	2	0	0
		Bradyrhizobium_diazoefficiens_USDA_110	1	2	1	1	1	2	1	4	1	2	0	0
		Bradyrhizobium_japonicum_USDA_6	1	4	1	1	1	2	1	3	0	2	0	0
		Bradyrhizobium_sp_BTAi1	1	2	1	1	1	1	0	1	0	2	1	0
		Bradyrhizobium_sp_ORS_278	1	4	1	1	0	1	0	1	2	1	1	0
		Brucella_abortus_S19	1	0	1	1	1	1	0	0	0	2	0	0
		Brucella_canis_ATCC_23365	1	1	1	1	1	1	0	0	0	2	0	1
		Brucella_melitensis_bv_1_str_16M	1	1	1	1	1	1	0	0	0	2	0	0
		Brucella_ovis_ATCC_25840	1	0	1	1	1	1	0	0	0	2	0	1
		Brucella_Suis_1330	1	2	1	1	1	1	0	0	0	2	0	1
		Candidatus_Liberibacter_asiaticus_str_psy62	0	0	1	0	0	0	0	0	0	1	0	0
		Candidatus_Liberibacter_solanacearum_CLso‐ZC1	0	0	1	0	0	0	0	0	0	1	0	0
		Candidatus_Midichloria_mitochondrii_IricVA	0	0	0	0	0	0	0	0	0	1	0	0
		Candidatus_Odyssella_thessalonicensis_L13	0	0	0	0	0	0	0	0	0	1	0	0
		Candidatus_Puniceispirillum_marinum_IMCC1322	1	0	1	1	1	1	0	2	0	0	0	0
		Chelativorans_sp_BNC1	1	0	1	1	1	1	0	0	0	1	0	0
		Fulvimarina_pelagi_HTCC2506	0	2	1	1	1	1	0	1	0	0	0	0
		Hoeflea_halophila_KCTC_23107	1	1	1	1	0	0	0	1	0	1	0	0
		Hoeflea_phototrophica_DFL‐43	1	1	1	1	0	0	0	1	0	1	0	0
		Hyphomicrobium_denitrificans_ATCC_51888	0	1	1	1	1	1	0	1	1	2	1	0
		Hyphomicrobium_sp_MC1	1	1	1	1	1	1	0	2	0	2	1	0
		Mesorhizobium_ciceri_biovar_biserrulae_WSM1271	1	2	1	1	3	2	0	2	1	2	1	0
		Mesorhizobium japonicum MAFF303099	1	0	1	1	2	1	2	2	0	3	1	0
		Mesorhizobium_loti_NZP2037	1	3	1	1	3	1	2	3	0	3	1	0
		Mesorhizobium_opportunistum_WSM2075	1	2	1	1	3	2	0	2	0	2	1	1
		Methylobacterium_extorquens_AM1	0	1	1	1	1	1	0	2	0	1	0	0
		Methylobacterium_extorquens_PA1	1	0	1	1	1	1	0	2	0	2	0	0
		Methylobacterium_nodulans_ORS_2060	1	2	1	1	1	1	1	3	0	3	0	0
		Methylobacterium_populi_BJ001	0	1	1	1	1	1	0	2	0	1	0	0
		Methylobacterium_radiotolerans_JCM_2831	2	0	1	1	1	1	0	2	0	2	1	0
		Methylobacterium_sp_4‐46	0	1	1	1	1	1	0	1	0	4	0	0
		Methylocella_silvestris_BL2	0	1	1	1	1	1	1	2	0	1	0	0
		Methylocystis_sp_ATCC_49242	0	1	1	1	1	1	0	0	0	0	0	0
		Methylosinus_trichosporium_OB3b	0	1	1	1	1	1	0	1	0	0	0	0
		Neorhizobium_galegae_HAMBI_1141	1	1	1	1	2	1	0	1	1	1	1	0
		Nitrobacter_hamburgensis_X14	0	0	1	1	1	1	0	1	0	1	0	0
		Nitrobacter_sp_Nb‐311A	0	1	1	1	1	1	0	1	0	2	0	0
		Nitrobacter_winogradskyi_Nb‐255	0	0	1	1	1	1	0	1	0	1	0	0
		Ochrobactrum_anthropi_ATCC_49188	0	0	1	1	1	1	0	0	0	2	0	0
		Oligotropha_carboxidovorans_OM5	1	1	1	1	1	1	0	3	0	1	0	0
		Parvibaculum_lavamentivorans_DS‐1	0	0	1	1	1	1	0	0	0	2	0	1
		Pelagibacterium_halotolerans_B2	1	2	1	1	1	1	0	0	0	1	0	0
		Rhizobium_etli_bv_mimosae_str_Mim1	1	1	1	1	2	1	1	2	2	3	1	0
		Rhizobium_etli_bv_phaseoli_str_IE4803	1	1	1	1	2	1	0	0	1	2	1	0
		Rhizobium_etli_CFN_42	1	1	1	1	2	1	0	1	2	2	1	0
		Rhizobium_etli_CIAT_652	1	1	1	1	2	1	0	0	1	2	1	0
		Rhizobium_gallicum_bv_gallicum_R602	1	1	1	1	1	1	1	2	1	2	1	0
		Rhizobium_leguminosarum_bv_phaseoli_CCGM1	1	1	1	1	2	1	0	0	1	2	1	0
		Rhizobium_leguminosarum_bv_trifolii_WSM2304	1	1	1	1	2	1	0	0	0	2	1	0
		Rhizobium_leguminosarum_bv_viciae_3841	1	2	1	1	2	1	0	1	1	2	1	0
		Rhizobium_leucaenae_USDA_9039	1	1	1	1	3	1	0	1	1	3	0	0
		Rhizobium_phaseoli_Brasil_5	1	1	1	1	2	1	0	2	1	2	1	0
		Rhizobium_tropici_CIAT_899	1	1	1	1	1	1	0	2	1	1	1	0
		Rhodomicrobium_vannielii_ATCC_17100	0	0	1	1	1	1	0	0	0	1	0	1
		Rhodopseudomonas_palustris_BisA53	0	0	1	1	1	1	0	2	0	2	0	0
		Rhodopseudomonas_palustris_BisB18	1	0	1	1	1	1	0	2	0	1	1	0
		Rhodopseudomonas_palustris_BisB5	1	0	1	1	1	1	0	3	0	2	0	0
		Rhodopseudomonas_palustris_CGA009	1	1	1	1	1	1	0	3	0	2	1	0
		Rhodopseudomonas_palustris_DX‐1	1	1	1	1	1	1	0	2	0	2	1	0
		Rhodopseudomonas_palustris_HaA2	1	0	1	1	1	1	0	3	0	2	1	0
		Sinorhizobium_fredii_HH103	1	1	1	1	2	1	1	0	2	3	1	0
		Sinorhizobium_fredii_NGR234	1	0	1	1	2	1	1	0	0	3	1	0
		Sinorhizobium_medicae_WSM419	1	0	1	1	2	1	0	0	0	1	1	0
		Sinorhizobium_meliloti_1021	1	0	1	1	2	1	0	0	0	2	1	0
		Starkeya_novella_DSM_506	1	3	1	1	1	1	0	1	0	1	0	0
		Xanthobacter_autotrophicus_Py2	1	0	1	1	1	1	1	1	0	2	1	0
	*Rhodobacterales*	Ahrensia_sp_R2A130	0	2	1	1	0	0	0	0	1	1	0	0
		Citreicella_sp_SE45	1	1	1	1	1	1	0	1	0	0	0	0
		Dinoroseobacter_shibae_DFL_12	1	0	1	1	1	1	0	1	0	1	0	0
		Hirschia_baltica_ATCC_49814	0	0	1	1	1	1	1	0	0	1	0	0
		Hyphomonas_neptunium_ATCC_15444	0	0	1	1	1	1	1	0	0	1	0	1
		Jannaschia_sp_CCS1	0	1	1	1	0	0	0	0	0	1	1	0
		Ketogulonigenium_vulgarum_WSH‐001	1	2	1	1	0	0	0	1	0	0	0	0
		Labrenzia_aggregata_IAM_12614	1	2	1	1	1	1	0	1	1	3	1	1
		Labrenzia_alexandrii_DFL‐11	1	2	1	1	1	1	0	0	0	1	0	1
		Maricaulis_maris_MCS10	0	0	1	1	1	1	1	0	0	0	0	0
		Maritimibacter_alkaliphilus_HTCC2654	1	1	1	1	1	1	1	1	0	0	0	0
		Oceanibulbus_indolifex_HEL‐45	1	2	1	1	0	0	0	1	0	0	1	0
		Oceanicaulis_sp_HTCC2633	0	0	1	1	1	1	1	0	1	0	0	0
		Oceanicola_batsensis_HTCC2597	1	1	1	1	1	1	0	1	0	0	0	0
		Oceanicola_granulosus_HTCC2516	1	1	1	1	1	1	0	1	0	1	0	0
		Octadecabacter_antarcticus_238	1	1	1	1	0	0	0	0	0	0	1	0
		Octadecabacter_antarcticus_307	1	2	1	1	0	0	0	0	0	0	1	0
		Paracoccus_denitrificans_PD1222	2	2	1	1	1	1	0	1	0	1	1	0
		Paracoccus_sp_TRP	1	0	1	1	1	1	0	0	0	1	1	0
		Pelagibaca_bermudensis_HTCC2601	0	2	1	1	1	1	0	1	2	2	0	0
		Phaeobacter_gallaeciensis_DSM_17395	1	1	1	1	1	1	0	0	0	1	0	0
		Pseudovibrio_sp_FO‐BEG1	1	1	1	1	1	1	0	0	0	2	0	1
		Rhodobacteraceae_bacterium_HTCC2083	1	2	1	1	0	0	0	0	0	0	0	0
		Rhodobacteraceae_bacterium_HTCC2150	0	1	1	1	1	1	0	0	0	0	0	0
		Rhodobacteraceae_bacterium_KLH11	2	1	1	1	1	1	0	0	0	0	1	0
		Rhodobacterales_bacterium_HTCC2255	1	1	1	1	1	1	0	0	0	0	0	0
		Rhodobacterales_bacterium_Y4I	1	2	1	1	1	1	0	0	0	2	0	0
		Rhodobacter_capsulatus_SB_1003	0	1	1	1	1	1	0	0	0	2	1	0
		Rhodobacter_sphaeroides_241	1	1	1	1	1	1	0	1	0	0	0	0
		Rhodobacter_sphaeroides_ATCC_17025	1	1	1	1	1	1	0	0	0	0	0	0
		Rhodobacter_sp_SW2	1	1	1	1	1	1	0	0	0	1	1	0
		Roseibium_sp_TrichSKD4	1	1	1	1	1	1	0	1	1	1	1	0
		Roseobacter_denitrificans_OCh_114	1	0	1	1	1	1	0	1	0	0	1	0
		Roseobacter_litoralis_Och_149	1	2	1	1	1	1	0	1	1	1	0	0
		Roseobacter_sp_AzwK‐3b	1	1	1	1	1	1	0	0	0	0	0	0
		Roseobacter_sp_CCS2	1	1	1	1	1	1	0	1	0	0	0	0
		Roseobacter_sp_GAI101	1	1	1	1	0	0	0	2	0	0	1	0
		Roseobacter_sp_MED193	1	1	1	1	1	1	0	1	1	2	0	0
		Roseobacter_sp_SK209‐2‐6	2	1	1	1	2	1	0	0	1	1	0	0
		Roseovarius_nubinhibens_ISM	1	2	1	1	1	1	0	0	0	0	0	0
		Roseovarius_sp_217	1	1	1	1	1	1	0	1	0	0	1	0
		Roseovarius_sp_TM1035	1	1	1	1	1	1	0	0	0	0	1	0
		Ruegeria_pomeroyi_DSS‐3	1	0	1	1	1	1	0	2	0	2	1	0
		Ruegeria_sp_R11	1	1	1	1	1	1	0	0	0	1	0	0
		Ruegeria_sp_TM1040	1	0	1	1	1	1	0	0	0	0	1	0
		Sagittula_stellata_E‐37	1	3	1	1	1	1	0	3	0	1	1	0
		Silicibacter_sp_TrichCH4B	2	2	1	1	1	1	0	1	0	0	1	0
		Sulfitobacter_sp_EE‐36	1	0	1	1	1	1	0	1	0	0	1	0
		Thalassiobium_sp_R2A62	1	1	1	1	1	1	0	0	0	0	0	0
	*Rickettsiales*	Anaplasma_centrale_str_Israel	0	0	1	1	1	1	0	0	0	1	0	0
		Anaplasma_marginale_str_Florida	0	0	1	1	1	1	0	0	0	1	0	0
		Anaplasma_marginale_str_St_Maries	0	0	1	1	1	1	0	0	0	1	0	0
		Anaplasma_phagocytophilum_HZ	0	0	1	0	0	0	0	0	0	0	0	0
		Ehrlichia_canis_str_Jake	0	0	1	0	0	0	0	0	0	1	0	0
		Ehrlichia_chaffeensis_str_Arkansas	0	0	1	0	0	0	0	0	0	1	0	0
		Ehrlichia_ruminantium_str_Welgevonden	0	0	1	0	0	0	0	0	0	1	0	0
		Neorickettsia_risticii_str_Illinois	0	0	1	0	0	0	0	0	0	1	0	0
		Neorickettsia_sennetsu_str_Miyayama	0	0	1	0	0	0	0	0	0	1	0	0
		Orientia_tsutsugamushi_str_Boryong	0	0	0	0	0	0	0	0	0	0	0	0
		Rickettsia_akari_str_Hartford	0	0	0	0	0	0	0	0	0	0	0	0
		Rickettsia_bellii_OSU_85‐389	0	0	0	0	0	0	0	0	0	0	0	0
		Rickettsia_canadensis_str_McKiel	0	0	0	0	0	0	0	0	0	0	0	0
		Rickettsia_conorii_Malish_7	0	0	0	0	0	0	0	0	0	0	0	0
		Rickettsia_endosymbiont_of_Ixodes_scapularis	0	0	0	0	0	0	0	0	0	0	0	0
		Rickettsia_felis_URRWXCal2	0	0	0	0	0	0	0	0	0	0	0	0
		Rickettsia_prowazekii_str_Madrid_E	0	0	0	0	0	0	0	0	0	0	0	0
		Rickettsia_sibirica_246	0	0	0	0	0	0	0	0	0	0	0	0
		Rickettsia_typhi_str_Wilmington	0	0	0	0	0	0	0	0	0	0	0	0
		Wolbachia_sp_wRi	0	0	1	0	0	0	0	0	0	0	0	0
	*Sphingomonadales*	Blastomonas_sp_RAC04	0	1	1	1	1	1	0	1	0	1	0	0
		Citromicrobium_bathyomarinum_JL354	0	0	1	1	1	1	0	0	0	0	0	0
		Citromicrobium_sp_JLT1363	0	0	1	1	1	1	0	0	0	0	1	0
		Novosphingobium_aromaticivorans_DSM_12444	0	0	1	1	1	1	0	2	0	1	1	0
		Novosphingobium_capsulatum_NBRC_12533	0	0	1	1	1	1	0	2	0	2	1	0
		Novosphingobium_nitrogenifigens_DSM_19370	0	0	1	1	1	1	0	0	0	2	1	0
		Novosphingobium_sp_PP1Y	1	1	1	1	1	1	0	1	0	1	0	0
		Sphingobium_chlorophenolicum_L‐1	1	1	1	1	1	1	0	0	0	1	1	0
		Sphingobium_japonicum_UT26S	0	1	1	1	1	1	0	0	0	1	0	0
		Sphingobium_sp_SYK‐6	0	1	1	1	1	1	0	1	0	0	1	0
		Sphingobium_yanoikuyae_XLDN2‐5	0	1	1	1	1	1	0	0	0	0	0	0
		Sphingomonas_paucimobilis_NBRC_13935	0	1	1	1	1	1	0	0	0	1	0	0
		Sphingomonas_sp_S17	0	1	1	1	1	1	0	0	0	1	0	0
		Sphingomonas_sp_SKA58	0	1	1	1	1	1	0	0	0	1	0	0
		Sphingomonas_wittichii_RW1	0	0	1	1	1	1	0	2	0	1	1	0
		Sphingopyxis_alaskensis_RB2256	0	0	1	1	1	1	0	0	0	2	0	0
		Sphingopyxis_macrogoltabida_strain_203	0	0	1	1	1	1	0	0	1	2	0	0
		Zymomonas_mobilis_subsp_mobilis_ATCC_10988	0	1	0	1	1	1	0	0	0	0	0	0
		Zymomonas_mobilis_subsp_pomaceae_ATCC_29192	0	1	0	1	0	0	0	0	0	0	0	0
	*Rhodospirillares*	Acetobacter_pasteurianus_IFO_3283‐01	1	0	1	1	1	1	0	2	0	2	1	0
		Acetobacter_pomorum_DM001	0	1	1	1	1	1	0	1	0	1	0	0
		Acidiphilium_cryptum_JF‐5	1	0	1	1	1	1	0	2	0	1	1	0
		Azospirillum_brasilense_Sp245	1	1	1	1	1	1	1	2	0	2	1	0
		Azospirillum_lipoferum_4B	2	2	1	1	1	1	0	2	0	1	1	0
		Azospirillum_sp_B510	2	0	1	1	1	1	0	2	0	0	1	0
		Enhydrobacter_aerosaccus_SK60	0	0	1	1	1	1	1	1	1	1	0	0
		Erythrobacter_litoralis_HTCC2594	0	0	1	1	1	1	0	0	0	0	0	1
		Erythrobacter_sp_NAP1	0	0	1	1	1	1	0	0	0	0	0	1
		Erythrobacter_sp_SD‐21	0	0	1	1	1	1	0	0	0	0	0	0
		Gluconacetobacter_diazotrophicus_PAl_5	1	2	1	1	1	1	0	1	0	2	1	0
		Gluconacetobacter_hansenii_ATCC_23769	1	1	1	1	1	1	0	1	0	1	1	0
		Gluconacetobacter_sp_SXCC‐1	1	2	1	1	1	1	0	1	0	2	1	0
		Gluconacetobacter_xylinus_NBRC_3288	1	2	1	1	0	1	0	0	0	2	0	0
		Gluconobacter_oxydans_621H	0	0	1	1	0	1	0	0	0	0	0	0
		Granulibacter_bethesdensis_CGDNIH1	1	0	1	1	1	1	0	1	0	1	0	1
		Magnetospirillum_magneticum_AMB‐1	0	0	1	1	1	1	1	1	0	1	0	0
		Rhodospirillum_rubrum_ATCC_11170	0	0	1	1	1	1	0	1	0	1	0	0
	*Caulobacterales*	Asticcacaulis_biprosthecum_C19	0	1	1	2	1	1	1	1	0	1	1	0
		Asticcacaulis_excentricus_CB_48	0	1	1	2	1	1	1	1	0	1	0	0
		Brevundimonas_diminuta_ATCC_11568	0	1	1	1	0	0	0	0	0	1	0	0
		Brevundimonas_sp_BAL3	0	1	1	2	1	1	1	0	0	0	0	0
		Brevundimonas_subvibrioides_ATCC_15264	0	1	1	2	1	1	1	1	0	1	0	0
		Brevundimonas_vesicularis_FDAARGOS_289	0	1	1	2	1	1	1	0	0	0	0	0
		Caulobacter_crescentus_CB15	0	0	1	1	1	1	1	0	0	2	0	0
		Caulobacter_mirabilis_FWC_38	0	1	1	1	1	1	1	0	0	1	0	0
		Caulobacter_segnis_ATCC_21756	0	1	1	1	1	1	1	0	0	2	0	0
		Caulobacter_sp_K31	0	0	1	1	1	1	1	1	0	2	0	0
		Phenylobacterium_zucineum_HLK1	0	0	1	1	1	1	1	0	1	0	1	1
	*Magnetococcales*	Loktanella_vestfoldensis_SKA53	1	2	1	1	0	0	0	1	0	0	0	0
		Magnetococcus_marinus_MC‐1	0	0	1	1	1	1	0	0	0	1	0	0
	*Parvularculales*	Parvularcula_bermudensis_HTCC2503	0	1	1	1	1	1	1	0	0	0	0	0
	*Pelagibacterales*	Candidatus_Pelagibacter_sp_HTCC7211	0	1	1	1	1	1	0	0	0	0	0	0
		Candidatus_Pelagibacter_sp_IMCC9063	0	1	1	1	1	1	0	0	0	0	0	0
		Candidatus_Pelagibacter_ubique_HTCC1062	0	0	1	1	1	1	0	0	0	0	0	0
			PYD3	PYD2	PYD1	KAR	KPHMT	PS	ADC	KPR	panZ	AAM	GabT	GAD
			108	123	187	183	167	170	35	44	28	137	68	14

**Table A3:** ADC phylogeny data set

Accession number	Organism	*Order*	Class
WP_0093402	*Afipia* sp. 1NLS2	*Rhizobiales*	α‐proteobacteria
NP_356949	*Agrobacterium fabrum* str. C58	*Rhizobiales*	α‐proteobacteria
WP_0416991	*Agrobacterium vitis*	*Rhizobiales*	α‐proteobacteria
WP_0027130	*Afipia clevelandensis*	*Rhizobiales*	α‐proteobacteria
WP_0123835	*Beijerinckia indica*	*Rhizobiales*	α‐proteobacteria
NP_768736	*Bradyrhizobium diazoefficiens* USDA 110	*Rhizobiales*	α‐proteobacteria
WP_0281441	*Bradyrhizobium japonicum* USDA 6	*Rhizobiales*	α‐proteobacteria
WP_0109135	(1)*Mesorhizobium japonicum* MAFF 303099	*Rhizobiales*	α‐proteobacteria
WP_0109160	*Mesorhizobium japonicum* MAFF 303099	*Rhizobiales*	α‐proteobacteria
WP_0198632	(1)*Mesorhizobium loti* NZP2037	*Rhizobiales*	α‐proteobacteria
WP_0198633	*Mesorhizobium loti* NZP2037	*Rhizobiales*	α‐proteobacteria
WP_0159320	*Methylobacterium nodulans*	*Rhizobiales*	α‐proteobacteria
WP_0125900	Methylocella_silvestris	*Rhizobiales*	α‐proteobacteria
WP_0209190	*Rhizobium etli* bv. Mimosae str. Mim1	*Rhizobiales*	α‐proteobacteria
WP_0401142	*Rhizobium gallicum*	*Rhizobiales*	α‐proteobacteria
WP_0143319	*Sinorhizobium fredii* HH103	*Rhizobiales*	α‐proteobacteria
YP_0028234	*Sinorhizobium fredii* NGR234	*Rhizobiales*	α‐proteobacteria
WP_0121143	*Xanthobacter autotrophicus*	*Rhizobiales*	α‐proteobacteria
WP_0062710	*Asticcacaulis biprosthecium*	*Caulobacterales*	α‐proteobacteria
WP_0134778	*Asticcacaulis excentricus*	*Caulobacterales*	α‐proteobacteria
WP_0082631	*Brevundimonas* sp. BAL3	*Caulobacterales*	α‐proteobacteria
WP_0132701	*Brevundimonas subvibrioides*	*Caulobacterales*	α‐proteobacteria
WP_0666264	*Brevundimonas vesicularis*	*Caulobacterales*	α‐proteobacteria
NP_421098	*Caulobacter crescentus* CB15	*Caulobacterales*	α‐proteobacteria
WP_0996228	*Caulobacter mirabilis*	*Caulobacterales*	α‐proteobacteria
WP_0109201	*Caulobacter segnis* ATCC 21756	*Caulobacterales*	α‐proteobacteria
WP_0122875	*Caulobacter* sp. K31	*Caulobacterales*	α‐proteobacteria
WP_0125231	*Phenylobacterium zucineum*	*Caulobacterales*	α‐proteobacteria
WP_0133008	*Parvularcula bermudensis*	*Parvularculales*	α‐proteobacteria
EEV21831.1	*Enhydrobacter aerosaccus* SK60	*Rhodospirillales*	α‐proteobacteria
WP_0141979	*Azospirillum brasilense*	*Rhodospirillales*	α‐proteobacteria
WP_0113863	*Magnetospirillum magneticum*	*Rhodospirillales*	α‐proteobacteria
WP_0158280	*Hirschia baltica*	*Rhodobacterales*	α‐proteobacteria
WP_0116463	*Hyphomonas neptunium* ATCC 15444	*Rhodobacterales*	α‐proteobacteria
WP_0116440	*Maricaulis maris*	*Rhodobacterales*	α‐proteobacteria
WP_0098031	*Oceanicaulis* sp. HTCC2633	*Rhodobacterales*	α‐proteobacteria
WP_0083357	*Maritimibacter alkaliphilus*	*Rhodobacterales*	α‐proteobacteria
YP_224431	*Corynebacterium glutamicum* ATCC 13032	*Corynebacteriales*	ε‐proteobacteria
WP_0108978	*Bacillus halodurans* c‐125	*Bacillales*	ε‐proteobacteria
NP_414673	*Escherichia coli* str. K‐12 substr. MG1655	*Enterobacterales*	γ‐proteobacteria
ABG68179.1	*Escherichia coli* 536	*Enterobacterales*	γ‐proteobacteria
NP_459185	*Salmonella enterica* subsp. enterica serovar Typhimurium str. LT2	*Enterobacterales*	γ‐proteobacteria
F6FYI9|F6F	*Ralstonia solanacearum* Po82	*Burkhorderiales*	β‐proteobacteria
NP_880521	*Bordetella pertussis Tohama* I	*Burkhorderiales*	β‐proteobacteria
NP_253419	*Pseudomonas aeruginosa* PAO1	*Pseudomonadales*	β‐proteobacteria
